# Unveiling Oral Malignant Melanoma: Clinicopathological Details of a Case

**DOI:** 10.7759/cureus.65101

**Published:** 2024-07-22

**Authors:** Saptarshi Das, Rojina Pervin, Sanjeet K Das, Arunit Chatterjee, Sudeshna Bagchi

**Affiliations:** 1 Oral and Maxillofacial Pathology, Guru Nanak Institute of Dental Sciences and Research, Kolkata, IND

**Keywords:** immunohistochemistry(ihc), metastasis, invasive pattern, melanocytes, oral malignant melanoma

## Abstract

Oral malignant melanoma is a rare tumor of the oral cavity. It is more common among Negros and Japanese people. Initial symptoms are frequently overlooked, resulting in a delayed diagnosis and poor prognosis with a 5-year survival rate. Unlike melanomas in other sites, it is uncommon and thus lacks a well-defined classification system and treatment regimen. The survival rate is mainly correlated with early diagnosis and treatment. A 54-year-old male reported to our department with a de novo fast-growing exophytic proliferative pigmented lesion for six months. After proper radiographic analysis, an incisional biopsy was done which revealed the presence of nests and fascicles of pleomorphic spindle cells with hyperchromatic nuclei and abundant brown pigments rendering it a provisional diagnosis of oral malignant melanoma which was later confirmed by immunohistochemistry (IHC). PET-CT scan revealed widespread metastasis. This article stresses the importance of identification of initial symptoms which are frequently overlooked, resulting in a delayed diagnosis and poor prognosis.

## Introduction

Malignant melanoma (MM) is a very aggressive tumor caused by the clonal growth and malignant transformation of melanocytes [1]. These are non-keratinocytes generated from neural crest cells, hence, malignant melanoma develops in any region where neural crest cells have migrated, including the mucosa. Melanoma is most commonly found in the skin, however, it can also appear in the oral mucosa. Oral malignant melanoma (OMM) is quite uncommon, ranging from 0.2 to 8% of all melanomas and 0.5% of all oral malignancies [2]. It typically appears as a pigmented macule or a quickly proliferating lesion in the maxillary gingiva/ridge or hard palate. Histologically abnormal melanocytes with hyperchromatism and nuclear pleomorphism, as well as immunohistochemical markers positive for S-100 and HMB-45, are considered confirmatory for the diagnosis of MM. The primary treatment for MM is radical excision with disease-free margins, however, recurrence occurs in approximately 20% of cases [3].

## Case presentation

A 54-year-old male patient reported to our department with the chief complaint of an irregular large soft tissue growth in the right edentulous maxillary region for the last six months. The lesion was small at first and gradually increased to the present size. When discomfort started, he visited a local clinician who referred him to our hospital. He had no history of long-standing pigmented oral lesion or deleterious oral habits and did not report any family history of similar disease. The teeth in the affected region were extracted long ago due to carious exposure and subsequent pain. Medical history revealed that the patient has been hypertensive for the past 10 years and is on oral drugs.

**Figure 1:**
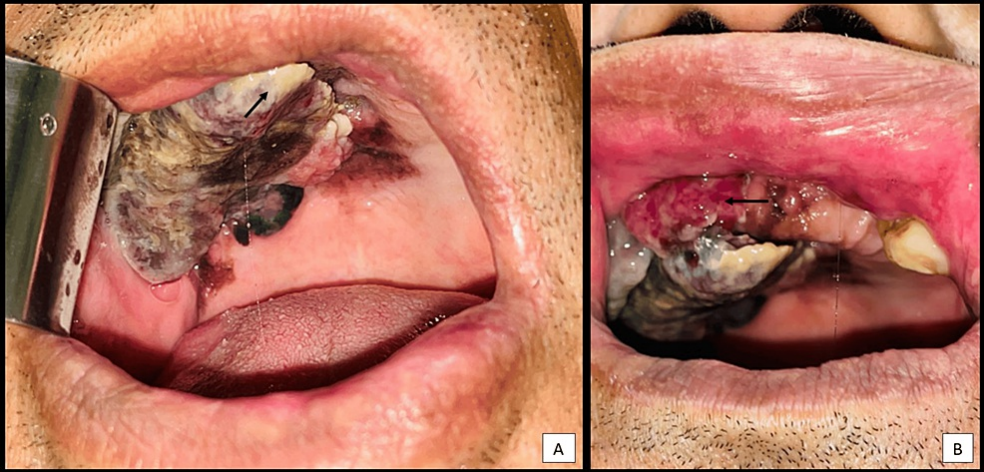
Irregular raised, exophytic, proliferative, brownish-black pigmented lesion covered with fibrinopurulent slough (A) with focal areas of haemorrhage (B) involving edentulous right half of the maxilla.

Intraoral examination revealed (Figure 1A, 1B) an irregular, raised, exophytic, proliferative, brownish-black pigmented lesion covered with fibrinopurulent slough with focal areas of haemorrhage measuring 4.8 x 4 cm involving edentulous right half of the maxilla, crossing the midline. On palpation, the lesion was soft to firm in consistency. Extraoral examination revealed bilateral cervical lymphadenopathy.

**Figure 2:**
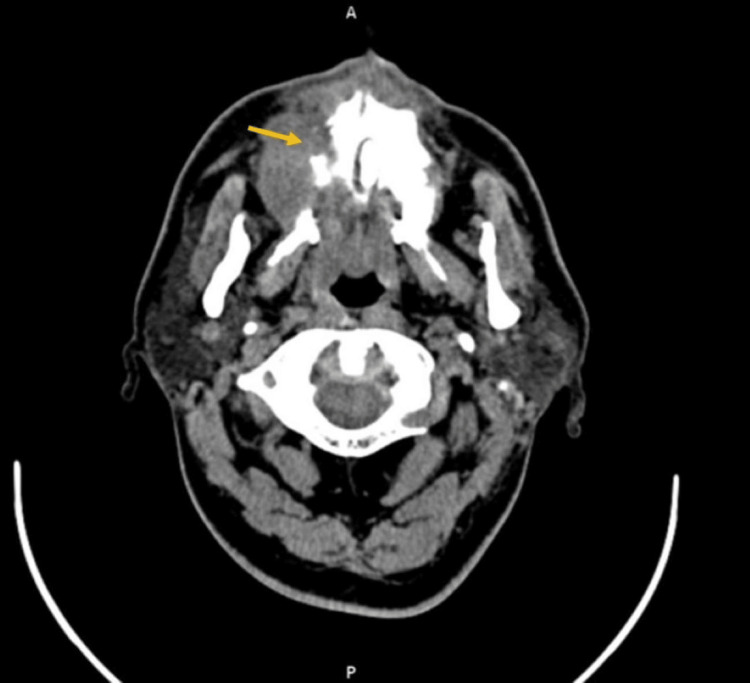
CECT revealed enhancing mass involving the right side of the palate. CECT = Contrast Enhanced Computerized Tomography

CECT of the area revealed (Figure 2) enhancing mass involving the right side of the palate and cervical lymphadenopathy.

Based on the history and clinical and radiological features, the diagnosis of an aggressive malignant growth was considered.

**Figure 3:**
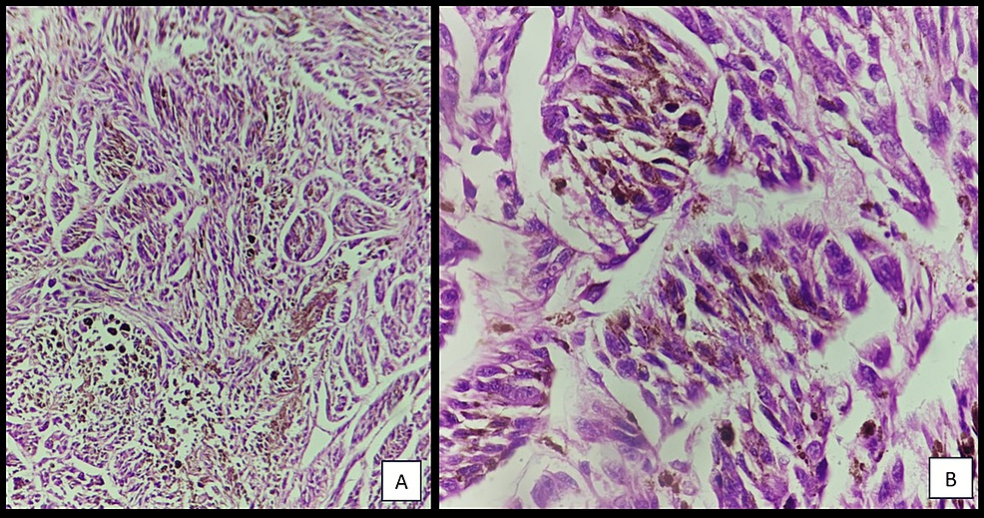
H&E stained sections revealed high-grade spindle cell neoplasm composed of pleomorphic brownish pigment-producing cells arranged in nest and fascicles infiltrating deep into the connective tissue stroma [(A) 10x, (B) 40x]. H&E = Hematoxylin and Eosin

An incisional biopsy was done after proper hematological investigations from the most representative area and was submitted for histopathological evaluation. The H&E stained sections revealed (Figure 3A, 3B) high-grade neoplasm being composed of pleomorphic spindle cells with hyperchromatic nuclei arranged in nest and fascicles infiltrating deep into the connective tissue stroma. There was the presence of abundant blackish-brown pigments in the cytoplasm of the cells.

**Figure 4:**
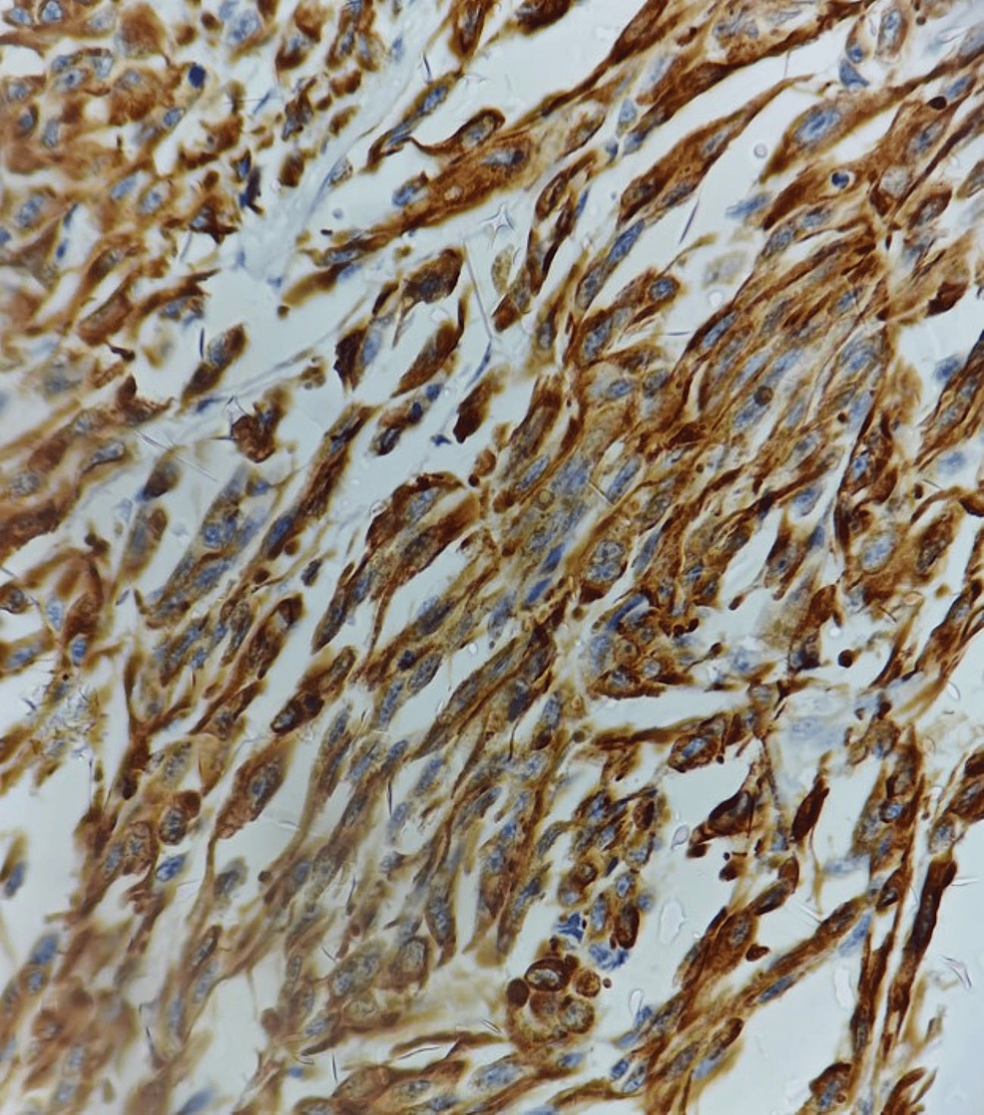
Immunohistochemistry showing strong reactivity of tumor cells to Vimentin [40x]

**Figure 5:**
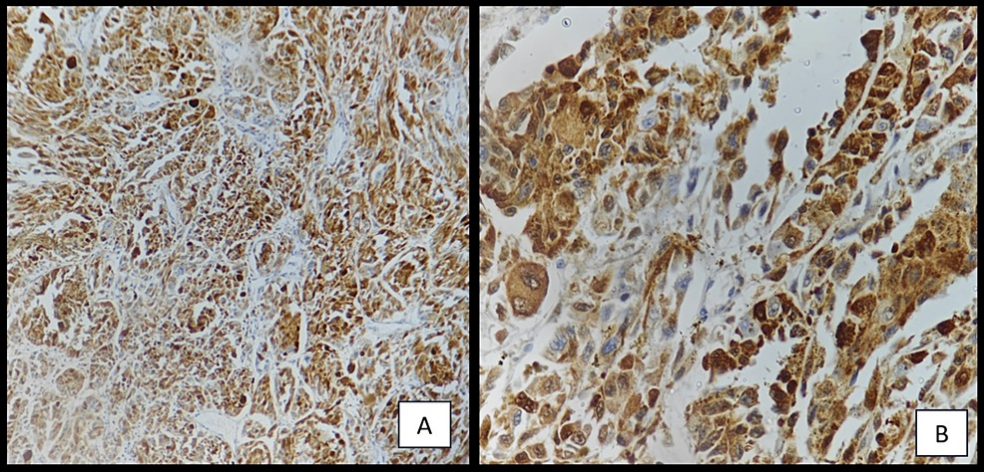
Immunohistochemistry showing strong reactivity of tumor cells to S 100 [(A) 10x, (B) 40x].

**Figure 6:**
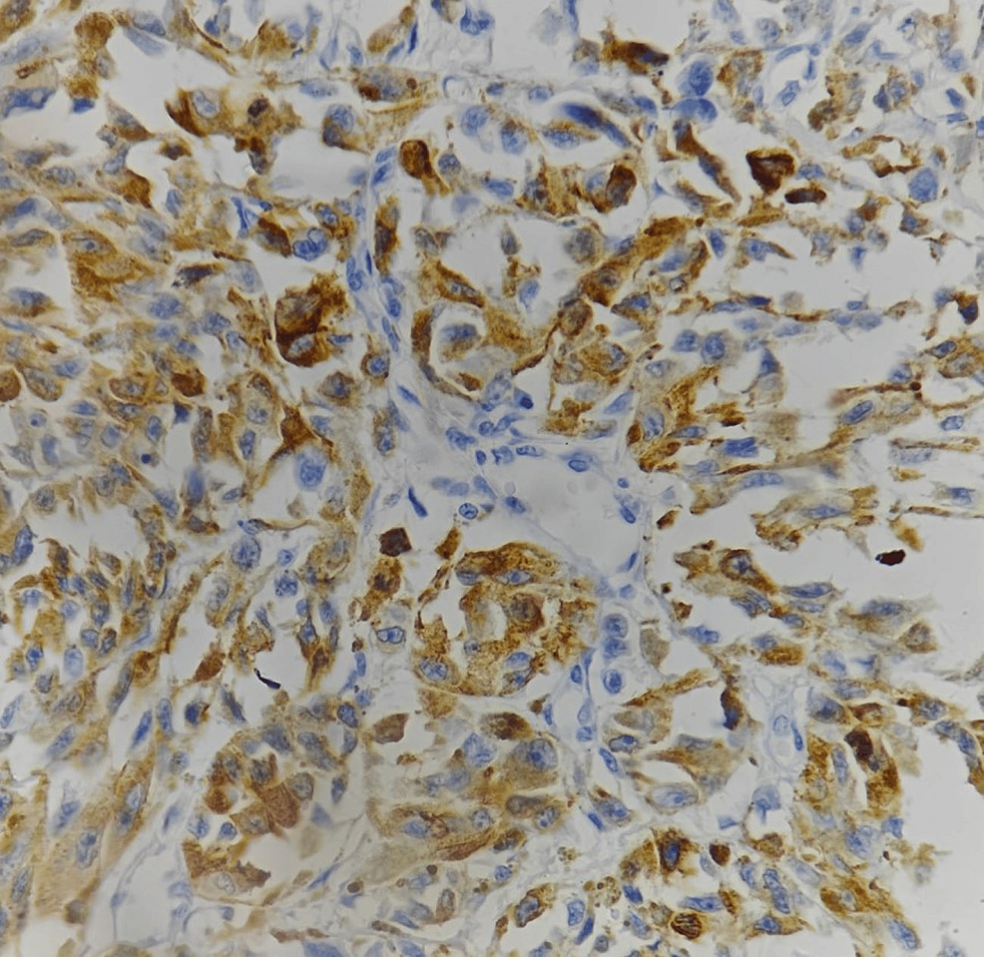
Immunohistochemistry showing strong reactivity of tumor cells to Melan A [40x]

**Figure 7:**
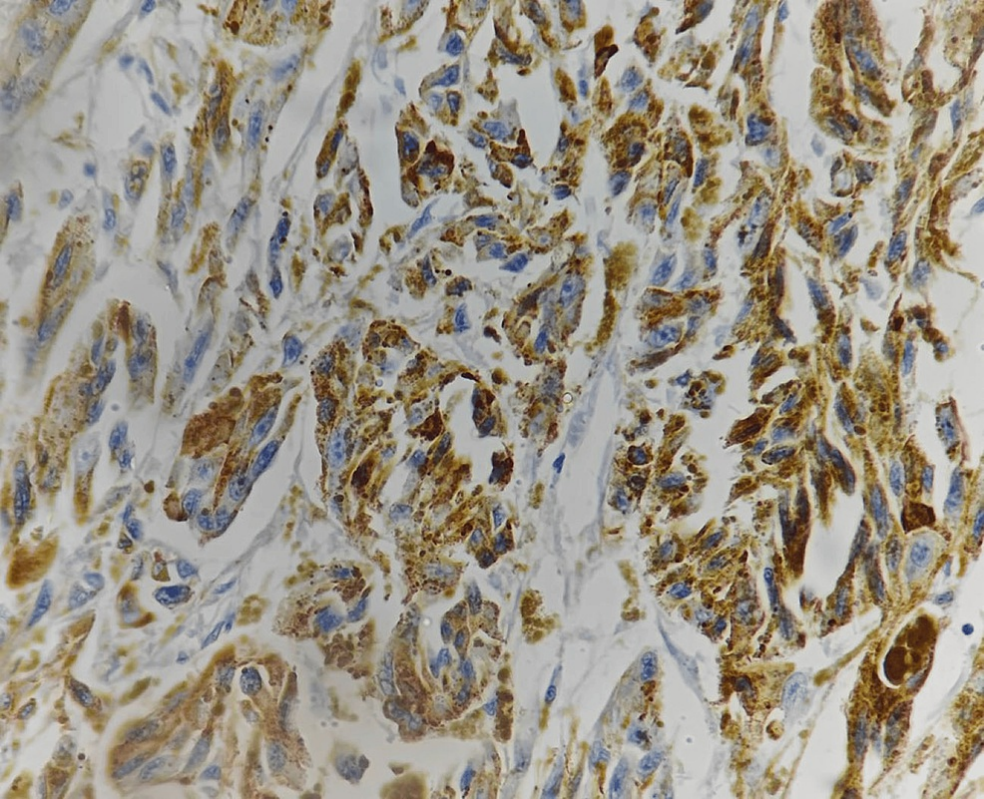
Immunohistochemistry showing strong reactivity of tumor cells to HMB 45 [40x]

**Figure 8:**
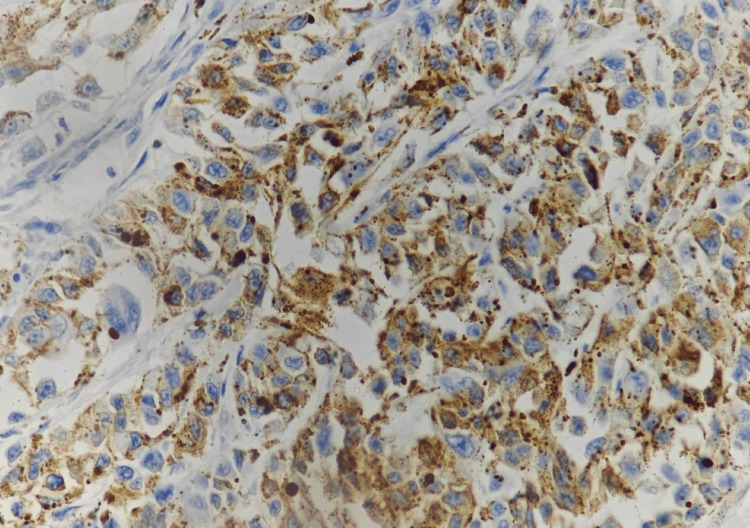
Immunohistochemistry showing negative nuclear staining with MITF of tumor cells [40x]

A provisional histopathological diagnosis of oral malignant melanoma was considered and to confirm the diagnosis, immunohistochemistry (IHC) was performed, which showed immunopositivity for Vimentin (Figure 4), S 100 (Figure 5A, 5B), Melan A (Figure 6), HMB 45 (Figure 7) and was immunonegative for MITF (Figure 8).

**Figure 9:**
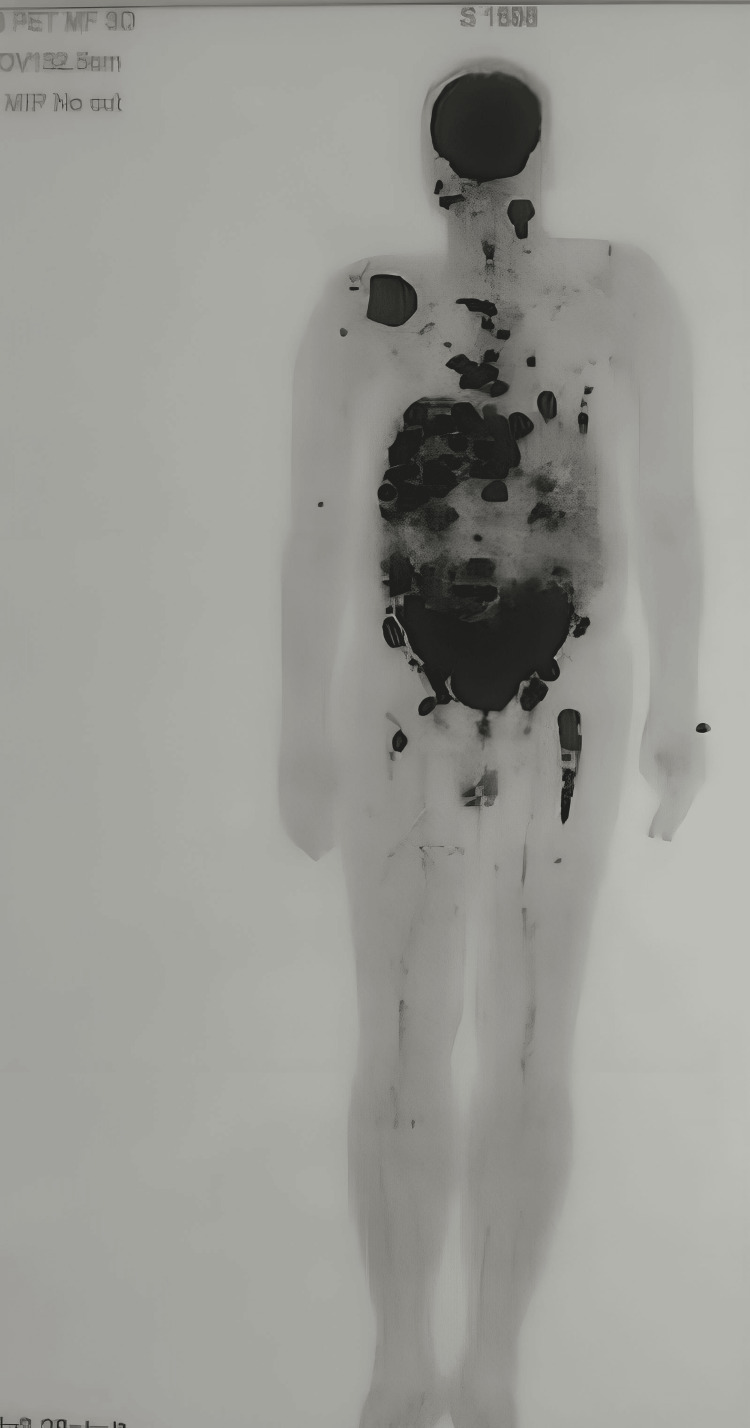
PET-CT showing metastatic foci in the parotid, submandibular salivary gland, subclavicular region, lungs, liver, spine, gastrointestinal region, bladder and focal areas of the femur PET-CT = Positron Emission Tomography-Computed Tomography

Positron emission tomography (PET) scans (Figure 9) revealed fluorodeoxyglucose (FDG) positive lesion involving the majority of the orofacial skeleton. The FDG-avid metastatic deposits were also observed in the parotid, submandibular salivary gland, subclavicular region, lungs, liver, spine, gastrointestinal region, bladder and focal areas of the femur.

The patient was referred to a medical oncology centre. The patient was lost to follow-up.

## Discussion

Mucosal melanomas comprise all oral melanomas along with those arising from mucous membranes of the conjunctiva, nose and pharynx. They penetrate and metastasize rapidly, and thus have a poorer prognosis than cutaneous melanomas [4].

Although excessive sun exposure and genetic factors (dysplastic nevus syndrome) have a role in the development of cutaneous MM, no known risk factors exist for mucosal MM [5,6]. However, smoking and long-term sensitivity to poorly fitting dentures have been suggested as potential risk factors [7]. Two-thirds of OMMs contain p53 protein changes, and a recent study found that loss of heterozygosity at 12p13 and loss of p27KIP1 protein expression [7] contribute to melanoma progression; whereas less than 10% of cases showed BRAF mutation [8]. Numerous studies have reported acral and mucosal melanoma to have overexpressed c-KIT (CD117) [9]. Melanocortin 1 receptor (MC1R) is a transmembrane G-protein-coupled receptor protein found in melanocytes which helps in DNA repair mechanisms. Stepwise elevation of MC1R expression has been noted in different stages of melanoma progression (nevi, primary, metastasis). Higher MC1R is also seen in deeper (>1 mm) primary lesions, ulcerated lesions, and mucosal melanomas compared to cutaneous melanomas and was associated with shorter survival in primary and metastatic tumors [10]. Involvement of other genes such as CDKN2A (p16), CDK4 (chromosome 12q15), RB I, CDKN2A (p19), PTEN/MMAC I and NRAS has also been observed [11].

Oral melanoma accounts for 0.2%-8.0% of all melanomas and 0.5% of all oral cancers. It is commonly seen in the 4th-6th decade of life, however, it is unusual before the 3rd decade. According to some authors, this has a 2:1 male predisposition [7]. OMM is thought to occur more commonly in Japanese, black, and Indian populations than whites [9]. The palate is the most typical location for oral melanomas. The gingiva, lips, floor of the mouth, and buccal mucosa are other, less frequent locations [4]. The case in this 54-year-old male patient discussed here was also located in the palate.

OMM frequently presents as a pigmented swelling. Typically, bleeding and ulceration appear when the lesion has progressed into the vertical development phase [7]. Malignant skin melanoma is categorized into four major types: 1. Lentigo maligna melanoma. 2. Superficial spreading melanoma. 3. Nodular melanoma. 4. Acral lentiginous melanoma [12]. Preexisting oral pigmented areas have been found in one-third of cases later diagnosed with melanoma [7]. OMM can be uniformly brown or black, or it can have varying pigmentation ranging from black to brown, grey, purple, red, and/or white. The lesions are asymmetric, irregular, and occasionally numerous, indicating satellite lesions [7]. Similarly in our patient, the lesion presented as a large, irregular, raised, exophytic, proliferative, brownish-black pigmented lesion which rapidly grew to the present size. According to Umeda et al., typical OMM usually has three components: a centrally placed nodule, a deep brownish-black plaque, and a light brown macular area [7]. Approximately 10% of cases are amelanotic, making diagnosis challenging [7]. Induration is typically absent in cases of protracted radial development phase. Other signs and symptoms include hemorrhage, pain or paresthesia, enhanced tooth mobility, and delayed healing of extraction sockets [7]. Tanaka et al. [7] distinguished five varieties of OMM by means of clinical presentation: the pigmented nodular, macular type, mixed type, nonpigmented nodular and mixed type. Therefore, it is crucial to rule out the possibility of primary malignant melanoma in another part of the body as the oral lesion can also be metastatic. Greene et al. described the following criteria for diagnosing primary OMM: 1. Presence of clinical and microscopic tumors in the oral mucosa. 2. Junctional activity detected in the oral mucosa. 3. Inability to display any further primary sites [7].

In spite of its aggressiveness and rapid metastatic spread to lymph nodes and hematogenous tissues, OMM can present as a flat, asymptomatic macule for a long time [13]. Unlike melanomas of other sites, the low incidence rate of OMM led to a lack of a well-defined classification system and treatment regimen. The ABCDEs criteria (asymmetry, border irregularity, color variegation, diameter, and evolving changes) that are commonly used to distinguish cutaneous malignant melanoma from other pigmented lesions can also be utilized to diagnose OMM [1]. Melanotic macule, smoker's melanosis, amalgam tattoo, Kaposi's sarcoma, and oral pigmented nevus are among the differential diagnoses [7].

Though incisional biopsy or other invasive operations have been proposed to cause tumor cell dispersion and metastasis, many experts believe that a biopsy should be performed because the benefits of an early and clear diagnosis outweigh the danger of distant metastasis [7]. According to Batsakis [7], “There is no evidence that a preliminary biopsy of the primary lesion increases the risk of metastatic dissemination or unfavorably affects prognosis”. Small lesions should be excised, while larger lesions should have incisional biopsy from the thickest and darkest part [7].

In addition to biopsies, radiologic testing using CT, MRI, or PET can be helpful in assessing the extent of the primary tumor invasion as well as any nearby or far-off metastasis, commonly seen in the lungs and liver, as seen in our case. A chest radiograph can be the most cost-effective primary tool to diagnose metastasis pre- and post-treatment [7].

According to the Western Society of Teachers of Oral Pathology (WESTOP), microscopically three patterns of mucosal melanomas have been identified: a) in situ pattern where the atypical pigmented melanocytes are restricted to the epithelial and connective tissue interface, b) invasive pattern where the tumor cells are discovered inside the supportive connective tissue and c) mixed pattern of invasive melanoma with an in situ component. The case discussed here shows the nest and fascicles of pleomorphic spindle cells having hyperchromatic nuclei infiltrating deep into the connective tissue stroma. The neoplastic cells can also be spindled, plasmacytoid, and epithelioid type and are usually arranged in sheetlike, organoid/alveolar, neurotropic, or desmoplastic manner [7].

There are no particular recommendations for OMM in the TNM staging of cutaneous melanoma. For head and neck mucosal melanomas, a clinical staging with a finer micro-staging approach (Table 1) has profound prognostic significance. Extensive metastasis has rendered our patient as stage III melanoma. The Breslow measurement (Table 2) is used to estimate tumor thickness, which is also a reliable indicator of prognosis [4].

**Table 1:** Clinical staging system for oral malignant melanoma with histopathologic microstaging for stage I.

Stages	Extension of the lesion
Stage I	Primary tumor present only (Tany N0 M0); Level I: Pure in situ melanoma without evidence of invasion or in situ melanoma with “microinvasion”, Level II: Invasion up to the lamina propria, Level III: Deep skeletal tissue invasion into skeletal muscle, bone or cartilage
Stage II	Tumor metastatic to regional lymph nodes (Tany N1 M0)
Stage III	Tumor metastatic to distant sites (Tany Nany M1)

**Table 2:** Breslow scale for the measurement of tumor thickness of oral malignant melanoma.

Thickness (mm)	Risk of recurrence
<0.76	Low risk
0.76 – 1.50	Low to intermediate risk
1.50 – 3.99	Intermediate to high risk
>4.00	High risk

Mucosal MMs have been found to be positive for 91%-95% of S-100 protein, 76%-98% of HMB-45, 78%-100% of tyrosinase, 65%-100% of Melan-A, and 57%-91% of microphthalmia-associated transcription factor or MITF [14]. S100 is the most sensitive melanoma marker but not specific [15], whereas, HMB 45 is much more specific. Still, 10%-15% of melanomas are negative to this marker [16]. The most effective stains for identifying desmoplastic mucosal MMs are tyrosinase and S-100 protein, although HMB-45 is negative in the majority of desmoplastic and spindle cell MMs [14]. The invasive phenotype is linked to low MITF activity and, thus, poorer prognosis [17]. Our case was immunopositive for vimentin, S 100, Melan A, HMB 45 and was immunonegative for MITF.

The preferred course of treatment for oral melanoma is radical surgery, but nowadays elective neck dissection is also being recommended [12]. Management of clinically negative nodes is still debatable. When treating inoperable tumors or elderly patients, radical surgery combined with chemotherapy and radiotherapy or radiotherapy alone is the chosen course of action. As an adjunct to surgical resection, immunochemotherapy and targeted therapy have demonstrated efficacy [12]. Targeted therapies interfere with the function of abnormal molecules within tumor cells regulating their growth. Commonly used targeted therapies used in melanoma are BRAF inhibitors like vemurafenib and dabrafenib; MEK inhibitors like trametinib and cobimetinib or C-KIT inhibitors like imatinib and nilotinib [18].

Oral lesions have a poorer prognosis than skin lesions; 31% of patients with localized illness survive for five years, compared to 5.2% in cases when cervical metastases are evident.

The poor prognosis associated with oral mucosal melanomas is more likely to be caused by the late presentation of patients with locally advanced disease. The mouth's high vascularity and lymphatic outflow promote early metastatic spread to nearby lymph nodes as well as farther away locations like the lungs and spinal column [12].

Early diagnosis is critical for successful therapy and may be the most important component in improving the prognosis of OMM.

## Conclusions

Oral malignant melanoma is a rare entity with a very high metastatic rate and thus, unfavourable prognosis. In this case report we have emphasized on various aspects of diagnostic procedures including CECT, PET CT, histopathology and immunohistochemistry which are the key aspects of early diagnosis and proper treatment planning, which in turn would help in better prognosis.

## References

[1] Gunasekaran N, Ajay A, Ahmed SP, Arumugam H (2023). Oral malignant melanoma - Clinical presentation with immunohistochemical analysis. J Cancer Res Ther.

[2] Manigandan T, Sagar GV, Amudhan A, Hemalatha VT, Babu NA (2014). Oral malignant melanoma: a case report with review of literature. Contemp Clin Dent.

[3] Misra SR, Tripathy UR, Das R, Mohanty N (2021). Oral malignant melanoma: a rarity!. BMJ Case Rep.

[4] Marx RE, Stern D (2003). Oral and Maxillofacial Pathology: A Rationale for Diagnosis and Treatment. Quintessence.

[5] Kousseff BG (1992). The genetics of malignant melanomas. Ann Plast Surg.

[6] Hoffman S, Yohn J, Robinson W, Norris D (1994). Melanoma: 1. Clinical characteristics. Hosp Pract (Off Ed).

[7] Mohan M, Sukhadia VY, Pai D, Bhat S (2013). Oral malignant melanoma: systematic review of literature and report of two cases. Oral Surg Oral Med Oral Pathol Oral Radiol.

[8] Thompson LD, Wieneke JA, Miettinen M (2003). Sinonasal tract and nasopharyngeal melanomas: a clinicopathologic study of 115 cases with a proposed staging system. Am J Surg Pathol.

[9] Warszawik-Hendzel O, Słowińska M, Olszewska M, Rudnicka L (2014). Melanoma of the oral cavity: pathogenesis, dermoscopy, clinical features, staging and management. J Dermatol Case Rep.

[10] Su DG, Djureinovic D, Schoenfeld D, Marquez-Nostra B, Olino K, Jilaveanu L, Kluger H (2024). Melanocortin-1 receptor expression as a marker of progression in melanoma. JCO Precis Oncol.

[11] Sivapathasundharam B (2016). Shafer’s Textbook of Oral Pathology, 8th ed.. Shafer’s Text Book of Oral Pathology. 8th ed. Chennai.

[12] Ram H, Mohammad S, Husain N, Devi S, Gupta PN (2010). Metastatic malignant melanoma of palate: a review of literature and report of an unusual case. Natl J Maxillofac Surg.

[13] Tchernev G, Lotti T, Wollina U (2018). Palatal melanoma: "the silent killer". Open Access Maced J Med Sci.

[14] Prasad ML, Jungbluth AA, Patel SG, Iversen K, Hoshaw-Woodard S, Busam KJ (2004). Expression and significance of cancer testis antigens in primary mucosal melanoma of the head and neck. Head Neck.

[15] Ferringer T (2011). Skin. Handbook of Practical Immunohistochemistry.

[16] Mahmood MN, Lee MW, Linden MD, Nathanson SD, Hornyak TJ, Zarbo RJ (2002). Diagnostic value of HMB-45 and anti-Melan A staining of sentinel lymph nodes with isolated positive cells. Mod Pathol.

[17] Hartman ML, Czyz M (2015). MITF in melanoma: mechanisms behind its expression and activity. Cell Mol Life Sci.

[18] (2024). Targeted therapy drugs for melanoma skin cancer. https://www.cancer.org/cancer/types/melanoma-skin-cancer/treating/targeted-therapy.html.

